# The cumulative impact of social determinants of health factors on mortality in adults with diabetes and chronic kidney disease

**DOI:** 10.1186/s12882-021-02277-2

**Published:** 2021-02-28

**Authors:** Mukoso N. Ozieh, Emma Garacci, Rebekah J. Walker, Anna Palatnik, Leonard E. Egede

**Affiliations:** 1 Department of Medicine, Division of Nephrology, Medical College of Wisconsin, Milwaukee, WI USA; 2 Center for Advancing Population Science, Medical College of Wisconsin, Milwaukee, WI USA; 3 Division of Nephrology, Clement J. Zablocki VA Medical Center, Milwaukee, WI USA; 4 Department of Medicine, Division of General Internal Medicine, Medical College of Wisconsin, Milwaukee, WI USA; 5 Department of Obstetrics and Gynecology, Medical College of Wisconsin, Milwaukee, WI USA

**Keywords:** Social determinants, Mortality, Kidney disease, Diabetes

## Abstract

**Background:**

A growing body of evidence supports the potential role of social determinants of health on health outcomes. However, few studies have examined the cumulative effect of social determinants of health on health outcomes in adults with chronic kidney disease (CKD) with or without diabetes. This study examined the cumulative impact of social determinants of health on mortality in U.S. adults with CKD and diabetes.

**Methods:**

We analyzed data from National Health and Nutrition Examination Surveys (2005–2014) for 1376 adults age 20 and older (representing 7,579,967 U.S. adults) with CKD and diabetes. The primary outcome was all-cause mortality. CKD was based on estimated glomerular filtration rate and albuminuria. Diabetes was based on self-report or Hemoglobin A1c of ≥6.5%. Social determinants of health measures included family income to poverty ratio level, depression based on PHQ-9 score and food insecurity based on Food Security Survey Module. A dichotomous social determinant measure (absence vs presence of ≥1 adverse social determinants) and a cumulative social determinant score ranging from 0 to 3 was constructed based on all three measures. Cox proportional models were used to estimate the association between social determinants of health factors and mortality while controlling for covariates.

**Results:**

Cumulative and dichotomous social determinants of health score were significantly associated with mortality after adjusting for demographics, lifestyle variables, glycemic control and comorbidities (HR = 1.41, 95%CI 1.18–1.68 and HR = 1.41, 95%CI 1.08–1.84, respectively). When investigating social determinants of health variables separately, after adjusting for covariates, depression (HR = 1.52, 95%CI 1.10–1.83) was significantly and independently associated with mortality, however, poverty and food insecurity were not statistically significant.

**Conclusions:**

Specific social determinants of health factors such as depression increase mortality in adults with chronic kidney disease and diabetes. Our findings suggest that interventions are needed to address adverse determinants of health in this population.

## Introduction

Chronic kidney disease (CKD) is a heterogeneous group of disorders characterized by alterations in kidney structure and function, which manifest as decreased glomerular filtration rate < 60 ml/min/1.73m^2^ or presence of urinary albumin excretion of ≥30 mg/day for at least 3 months [1]. CKD affects 15% of United States adults and is the 9th leading cause of death [2, 3]. Research published in the last two decades show an uneven burden of CKD and identifies disparities in its incidence and outcomes with minorities being at highest risk [4, 5]. A growing body of evidence supports the potential role of social determinants in explaining the CKD disparities [6–8].

Social determinants of health are defined by The World Health Organization as “conditions in which people are born, grow, work, live, and age” [9]. Social determinants are often categorized into four groups of interacting factors: 1) socioeconomic circumstances, 2) psychosocial factors, 3) neighborhood environment, and 4) political, economic and cultural drivers [10, 11]. These include factors such as food insecurity, housing instability, social support, and violence in one’s community [11, 12]. Evidence supports an association between social determinants and the incidence and prevalence of chronic disease [11]. Social determinants of health may influence health by mediating availability of resources to maintain health, access to healthcare, and modify risk of exposure to environmental hazards and stress [6].

The relationship between individual social determinants of health factors and outcomes in adults with CKD is gaining more attention. For instance, studies show that socioeconomic status, specifically poverty, is associated with CKD risk factors [13–15], CKD [16], CKD progression [17], incident end stage renal disease (ESRD) [18–20] and mortality [21]. Food insecurity, a neighborhood determinant, has been shown to be associated with CKD [22], CKD progression to ESRD [23] and mortality [24]. Depression, a psychosocial determinant, is associated with incident CKD in patients with diabetes [25], progression to ESRD and mortality in individuals with CKD with or without diabetes [26–29]. In a meta-analysis of 83,381 individuals, an association between depression and a higher risk of death in individuals with non-dialysis dependent CKD was established [27].

To our knowledge, no studies has examined the cumulative effect of social determinants of health factors on health outcomes in adults with CKD with or without diabetes. Therefore, in this study we sought to understand the cumulative and individual association between social determinants of health and mortality in a nationally representative sample of U.S. adults with CKD and diabetes.

## Methods

### Study design and population

The National Health and Nutrition Examination Survey (NHANES) is a program of studies designed to assess the health and nutritional status of adults and children in the United States [30]. The survey is unique in that it combines interviews and physical examinations.

This study used five cycles of NHANES data between the years 2005–2014. 2014 is the latest survey available with linked mortality from the National Death Index (NDI). The start date for each individual was the date they completed the NHANES survey. Death date was set at as the date included in the linked National Death Index data file and individuals who were not deceased were censored at the end of the study follow-up date (December 31st, 2015). The study population was limited to adults 20 years of age and older, who 1) participated both in the interview and physical examination, 2) had both CKD and diabetes, and 3) had linked mortality data from NDI. In total, 1652 participants were included in the analysis.

### Measurements and definitions

#### Diabetes definition

Diabetes was defined as self-reported diabetes, or a hemoglobin A1c (HbA1c) ≥ 6.5% per 2016 American Diabetes Association guidelines [31]. Self-reported diabetes were based on individuals answering “yes” to any of the following three questions: “Have you ever been told by a doctor or other health professional that you have diabetes or sugar diabetes?”, “Are you now taking insulin?”, or “Are you now taking diabetic pills to lower your blood sugar?”

#### Chronic kidney disease (CKD) definition

CKD was defined based on Estimated glomerular filtration rate (eGFR) categories (G1-G5) and albuminuria/urine albumin-to-creatinine ratio categories (A1-A3) according to the Kidney Disease Improving Global Outcomes (KDIGO) Clinical Practice Guidelines for the Evaluation and Management of Chronic Kidney Disease [1]. Urinary albumin and urinary creatinine were measured from a random urine sample collected in the Mobile Exam Center. Urine albumin-to-creatinine ratio was calculated based on the measured values. eGFR was calculated using the chronic kidney disease epidemiology equation, the recommended formulae for eGFR per the KDIGO guidelines [1]. Creatinine from the serum specimen collected in the Mobile Exam Center was used to calculate eGFR. The Jaffe rate method was used to determine the concentration of creatinine in serum for NHANES 2005–2006. As such, a correction of the NHANES 2005–2006 serum creatinine was done while no correction was required for NHANES 2007–2014 since serum creatinine were standardized for these years.

#### Mortality outcome

Mortality outcome of interest for this study was all-cause mortality. National Center for Health Statistics has linked various surveys with death certificate records from National Death Index (NDI) [32]. The public-use linked mortality file provides mortality follow-up data from the date of survey participation through December 31, 2015. Participants who were not matched with death records after the censor date for the study were considered to be alive and assigned the number of person months. All participants with sufficient identifying data were eligible for mortality follow-up.

#### Social determinants of health measures

Social determinants of health measures included family income to poverty ratio level, household food insecurity, and depression.

- Family Income to Poverty Ratio: The family income to poverty ration was calculated by dividing family income by the poverty guidelines specific to the survey year. The value was not computed if the respondent only reported income as <$20,000 or ≥ $20,000. We dichotomized the ratio into poor (≤130% of poverty level) and not poor (> 130% of poverty level) based on the income cut-off for a number of government programs.
- Food Insecurity: Food insecurity was measured using the U. S Food Security Survey Module which consists of 18 questions asked of households with children and 10 questions asked of households without children [33]. Four response levels were created based on the number of affirmative responses for these questions [33]:
  1. Household full food security: no affirmative response in any of these items.
  2. Household marginal food security: 1–2 affirmative responses.
  3. Household low food security: 3–5 affirmative responses for household without children under the age of 18; 3–7 affirmative responses for household with children
  4. Household very low food security: 6–10 affirmative responses for household without children under the age of 18; 8–18 affirmative responses for household with children.

We further dichotomized the four categories into two distinctive categories: food secure (full food security and marginal food security) and food insecure (low food security and very low food security).

c) Depression: Depression was measured using the Patient Health Questionnaire-9 (PHQ-9) which is a 9-item self-reported assessment of symptoms matching to the fourth edition of the Diagnostic and Statistical Manual of Mental Disorders assessment for depression [34]. The nine symptom questions are scored from “0” (not at all) to “3” (nearly every day). We dichotomized depression into no depression (PHQ-9 scores: 0–4) and depression (PHQ-9 scores: 5–27).

Cumulative social determinant score: We constructed a cumulative score by counting the number of the three social determinant measures present for each individual. The cumulative score ranged from 0 to 3, with 3 indicating adverse social determinant of health factors across the three domains. For this outcome we excluded participants that did not have scores for all three social determinant factors, so 1376 participants were used for the cumulative score analysis.

Dichotomous social determinant measure: A dichotomous social determinant measure was created using all three social determinant measures, with *“No adverse SDOH or 0”* representing absence of any adverse social determinant and *“Adverse SDOH or* ≥ *1”* representing presence of any adverse social determinant.

#### Covariates

Demographic variables included gender, age in years, race/ethnicity (grouped as non-Hispanic White; non-Hispanic Black; Hispanic; and other minority), education level was treated as an ordinal variable with five levels (<9th grade, 9 to 11th (includes 12th grade with no diploma, high school graduate/general education diploma or equivalent, some college or associates degree and college graduate or above) and insurance coverage (dichotomized as yes vs. no).

Lifestyle variables included: a) physical activity dichotomized as none (no physical activity) vs. moderate to vigorous physical activity, b) smoking status dichotomized as none smoker vs. former or current smoker and, c) drinking status dichotomized as none drinker vs. moderate or above moderate drinker.

Glycemic control measured using HbA1c level was included in the model as a continuous variable based on every 1% increase in HbA1c level.

Comorbidities were dichotomized as “presence” or “absence” of the following medical conditions: cancer, hypertension, heart disease and stroke.

### Statistical analyses

Statistical analysis was performed with SAS version 9.4 (SAS Institute) and accounted for the complex survey design using the SURVEYFREQ, SURVEYMEANS, and SURVEYPHREG procedures. A series of Survey Cox proportional hazards regression models were run to investigate the relationship between social determinants of health and all-cause mortality. First, we ran a univariate Cox model then we ran three multivariate Cox models in hierarchical sequence: 1) adjusting for demographic variables; 2) adjusting for demographic and lifestyle variables and; 3) adjusting for demographic, lifestyle, glycemic control and comorbidity variables. In the first set of models, the cumulative social determinant score was used as the primary independent variable. In the second set of models the dichotomous social determinant score was used as the primary independent variables. In the third set of models, the three individual social determinant measures (food insecurity, family income to poverty ratio, and depression) were entered together to investigate the independent effect of each on all-cause mortality. Missing value were treated as missing at random with the covariate missing percentage being < 0.5%. Statistical significance was based on *p* < 0.05.

## Results

Sample demographics for the weighted population are presented in Table 1. The mean age was 63.5 years, with 30.6% reporting poverty, 16.1% reporting food insecurity, 32.6% reporting depression and 53.4% reporting presence of any adverse social determinants. The majority of the sample was non-Hispanic White (63.2%) and had insurance coverage (89.5%).

**Table 1:** Weighted Sample Demographics for Adults with Chronic Kidney Disease and Diabetes

(*n* = 1376; *N* = 7,579,967)	Percentage
**Age in years at screening**	
20–54	25.8%
55–74	48.2%
75+	26.0%
**Gender**	
Male	51.7%
Female	48.3%
**Race**	
Non-Hispanic White	63.2%
Non-Hispanic Black	16.5%
Other	20.4%
**Education group**	
< 9th grade	14.1%
9-11th grade	16.3%
High school graduate/GED or equivalent	26.2%
Some college or associate degree	29.3%
College graduate or above	14.1%
**Marital status**	
Married	54.6%
not Married	45.4%
*Insurance coverage	
Private insurance	51.1%
Medicare	52.7%
Medicaid	13.1%
No Insurance	10.5%
**Lifestyle Factors**	
Former or current smoker	55.2%
Moderate or above moderate drinker	60.9%
No physical activity	55.3%
**Comorbidity**	
Hypertension	75.4%
Heart Disease	30.0%
Stroke	13.1%
Cancer	16.1%
**Social Determinant Measures**	
≤ 130% of poverty level	30.6%
Food insecurity	16.1%
Mild to Severe depression	32.6%
**Social Determinant Composite Score**	
No adverse social determinant score (0)	46.6%
Adverse social determinant score (≥1)	53.4%
**Mortality Status**	
Alive	76.7%
Deceased	23.3%

*Insurance coverage reported includes only major types

Table 2 provides the unadjusted and adjusted results for the relationship between a cumulative social determinant score and mortality. Prior to adjustment, the association between a cumulative social determinants of health score and mortality was not statistically significant with the hazard ratio at 1.11 (95%CI 0.97, 1.28). After adjusting for demographics, the hazard ratio became significant at 1.41 (95%CI 1.18, 1.67). This remained significant after adding lifestyle variables, glycemic control and comorbidities with hazard ratio of 1.41 (95%CI 1.18, 1.68), for every 1 score increase in the cumulative social determinant scale.

**Table 2:** Cox Proportional Hazard Model for Relationship of Cumulative Social Determinant of Health Score on Mortality

	Unadjusted		Adjusted for Demographics		Adjusted for Demographics and Lifestyle		Adjusted for Demographics, Lifestyle, Glycemic Control and Comorbidities	
	Hazard Ratio (95% CI)	***P***- Value	Hazard Ratio (95% CI)	***P***- Value	Hazard Ratio (95% CI)	***P***- Value	Hazard Ratio (95% CI)	***P***- Value
**SDOH cumulative score**	1.11 (0.97–1.28)	0.13	^a^1.41 (1.18–1.67)	< 0.01	^a^1.39 (1.18–1.64)	< 0.01	^a^1.41 (1.18–1.68)	< 0.01
**Gender** Female (ref Male)			^a^0.69 (0.51–0.94)	0.02	^a^0.61 (0.45–0.84)	< 0.01	^a^0.65 (0.46–0.91)	0.01
**Age at Screening** (Increase 1 year)			^a^1.06 (1.05–1.07)	< 0.01	^a^1.06 (1.04–1.07)	< 0.01	^a^1.05 (1.04–1.06)	< 0.01
**Race** (ref non-Hispanic White)				0.03		0.02		0.03
non-Hispanic Black			0.84 (0.61–1.14)		0.83 (0.61–1.12)		0.82 (0.60–1.11)	
Other			^a^0.66 (0.48–0.90)		^a^0.64 (0.47–0.88)		^a^0.65 (0.48–0.90)	
**Education level** (Increase 1 level)			0.93 (0.84–1.02)	0.13	0.97 (0.88–1.08)	0.60	1.00 (0.90–1.10)	0.93
**Insurance coverage** No (ref Yes)			0.67 (0.32–1.42)	0.29	0.74 (0.34–1.57)	0.42	0.69 (0.31–1.53)	0.36
**Lifestyle Factors**								
Physical activity Moderate to Vigorous (ref none)					^a^0.51 (0.38–0.69)	< 0.01	^a^0.52 (0.37–0.73)	< 0.01
Smoking status Former or Current Smoker (ref none)					^a^1.38 (1.08–1.76)	< 0.01	^a^1.35 (1.05–1.74)	0.02
Drinking status Moderate or above Drinker (ref none)					^a^0.61 (0.45–0.82)	< 0.01	^a^0.59 (0.43–0.82)	< 0.01
**Glycemic Control**								
Hemoglobin A1c (%) (Increase 1%)							^a^1.09 (1.01–1.18)	0.03
**Comorbidity**								
Hypertension (Ref No Hypertension)							1.00 (0.71–1.42)	1.00
Heart disease (Ref No Heart Disease)							^a^1.75 (1.35–2.32)	< 0.01
Stroke (Ref No Stroke)							0.99 (0.64–1.54)	0.96
Cancer (Ref No Cancer)							^a^1.45 (1.03–2.02)	0.03

^a^Indicates significant hazard ratio

**Fig. 1:**
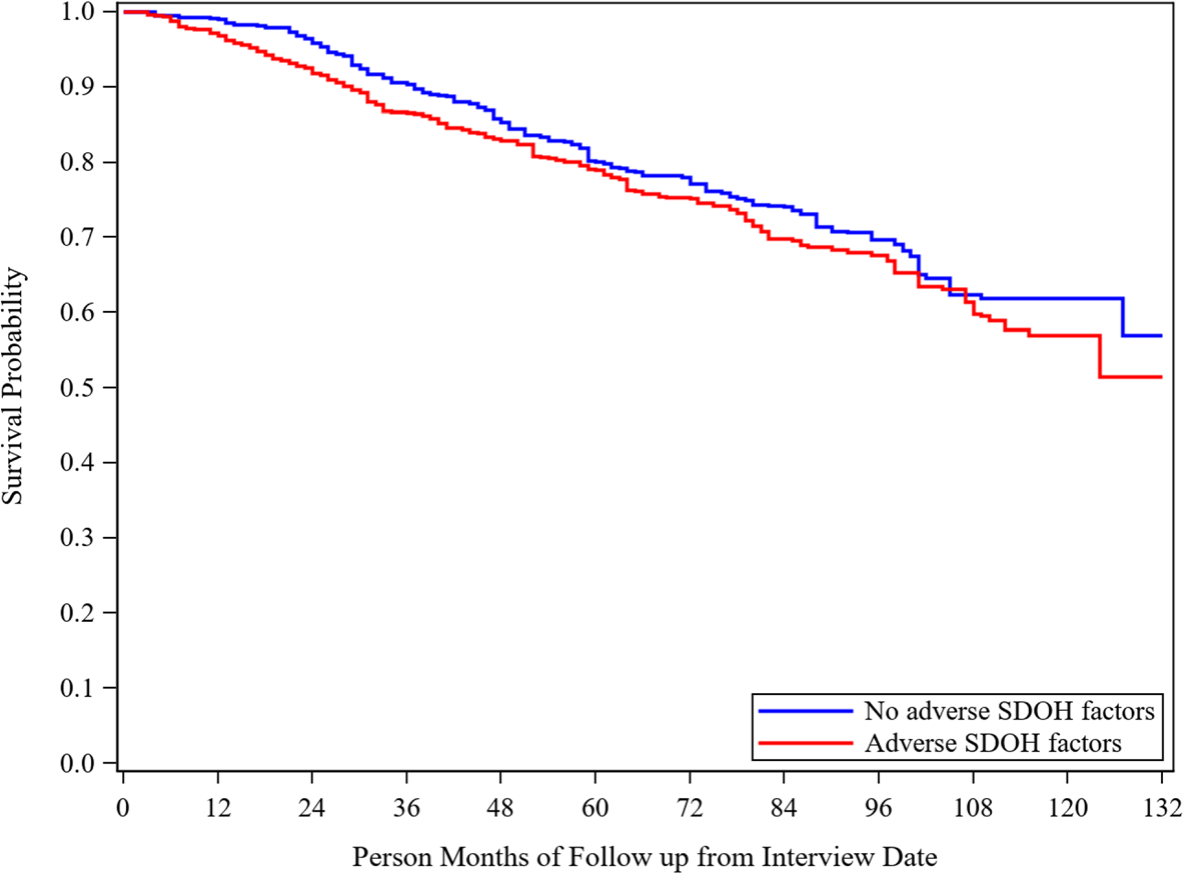
Weighted Kaplan-Meier Estimates for Relationship of Dichotomous Social Determinant of Health (SDOH) Score on Mortality

Table 3 and Fig. 1 provides the results of the association between the dichotomous social determinants variable and mortality. Similar results were found with the fully adjusted model indicating a significant association between presence of any adverse social determinants of health factor and mortality (HR = 1.41, 95%CI 1.08, 1.84).

**Table 3:** Cox Proportional Hazard Model for Relationship of Dichotomous Social Determinant of Health Score on Mortality

	Unadjusted		Adjusted for Demographics		Adjusted for Demographics and Lifestyle		Adjusted for Demographics, Lifestyle, Glycemic Control and Comorbidities	
	Hazard Ratio (95% CI)	***P***- Value	Hazard Ratio (95% CI)	***P***- Value	Hazard Ratio (95% CI)	***P***- Value	Hazard Ratio (95% CI)	***P***- Value
**Dichotomous SDOH score group**		0.19		0.01		0.02		0.01
No adverse SDOH factors (0)	Ref		Ref		Ref		Ref	
Adverse SDOH factors (≥1)	1.19 (0.91–1.56)		^a^1.42 (1.07–1.88)		^a^1.38 (1.05–1.80)		^a^1.41 (1.08–1.84)	
**Gender** Female (ref Male)			^a^0.72 (0.53–0.97)	0.03	0.64 (0.47–0.88)	< 0.01	^a^0.67 (0.48–0.93)	0.02
**Age at Screening** (Increase 1 year)			^a^1.06 (1.04–1.07)	< 0.01	^a^1.05 (1.04–1.06)	< 0.01	^a^1.05 (1.03–1.06)	< 0.01
**Race** (ref non-Hispanic White)				0.07		0.06		0.08
non-Hispanic Black			0.85 (0.62–1.16)		0.85 (0.62–1.15)		0.84 (0.61–1.16)	
Other			^a^0.69 (0.50–0.94)		^a^0.68 (0.49–0.93)		^a^0.69 (0.50–0.95)	
**Education level** (Increase 1 level)			0.90 (0.82–1.00)	0.04	0.95 (0.86–1.05)	0.31	0.97 (0.88–1.07)	0.57
**Insurance coverage** No (ref Yes)			0.71 (0.34–1.49)	0.36	0.78 (0.37–1.66)	0.52	0.75 (0.35–1.62)	0.46
**Lifestyle Factors**								
Physical activity Moderate to Vigorous (ref none)					^a^0.51 (0.38–0.69)	< 0.01	^a^0.52 (0.37–0.73)	< 0.01
Smoking status Former or Current Smoker					^a^1.40 (1.10–1.80)	< 0.01	^a^1.37 (1.05–1.79)	0.02
Drinking status Moderate or above drinker (ref none)					^a^0.62 (0.46–0.83)	< 0.01	^a^0.59 (0.43–0.82)	< 0.01
**Glycemic Control**								
Hemoglobin A1c (%) (Increase 1%)							^a^1.10 (1.01–1.19)	0.02
**Comorbidity**								
Hypertension (Ref No Hypertension)							1.02 (0.73–1.44)	0.90
Heart disease (Ref No Heart Disease)							^a^1.80 (1.37–2.37)	< 0.01
Stroke (Ref No Stroke)							0.97 (0.62–1.53)	0.91
Cancer (Ref No Cancer)							^a^1.41 (1.00–1.99)	0.05

^a^Indicates significant hazard ratio

Table 4 provides the results of incorporating all three social determinants of health measures as individual factors. Prior to adjustment, when the three variables were entered into the same model, only depression had a statistically significant association with mortality (depression HR = 1.33, 95%CI 1.04, 1.71). After adjusting for demographics, lifestyle variables, glycemic control and comorbidities, depression (HR = 1.41, 95%CI 1.10, 1.82) maintained statistical significance and was independently associated with mortality, however food insecurity (HR = 1.41, 95%CI 0.95, 2.07, *p*-value 0.09) and poverty (HR = 1.40, 95%CI 1.00–1.97) were not independent risk factors for mortality.

**Table 4:** Cox Proportional Hazard Model for Relationship of Individual Social Determinant of Health Measures on Mortality

	Unadjusted		Adjusted for Demographics		Adjusted for Demographics and Lifestyle		Adjusted for Demographics, Lifestyle, Glycemic Control and Comorbidities	
	Hazard Ratio (95% CI)	***P***- Value	Hazard Ratio (95% CI)	***P***- Value	Hazard Ratio (95% CI)	***P***- Value	Hazard Ratio (95% CI)	***P***- Value
**Ratio of family income to poverty group**		0.09		0.03		0.08		0.05
≤130% of poverty level (ref > 130% of poverty level)	1.29 (0.96–1.74)		^a^1.46 (1.04–2.05)		1.31 (0.97–1.79)		1.40 (1.00–1.97)	
**Household food security group**		0.05		0.17		0.06		0.09
Food insecure (ref Food secure)	0.70 (0.49–1.00)		1.27 (0.90–1.80)		1.42 (0.99–2.04)		1.41 (0.95–2.07)	
**Depression group**		0.02		< 0.01		< 0.01		< 0.01
Mild to Severe depression (ref No depression 0–4)	^a^1.33 (1.04–1.71)		^a^1.44 (1.12–1.86)		^a^1.45 (1.12–1.86)		^a^1.41 (1.10–1.82)	
**Gender** Female (ref Male)			^a^0.69 (0.51–0.93)	0.02	^a^0.61 (0.45–0.84)	< 0.01	^a^0.65 (0.46–0.91)	0.01
**Age at Screening** (Increase 1 year)			^a^1.06 (1.05–1.07)	< 0.01	^a^1.06 (1.04–1.07)	< 0.01	^a^1.05 (1.04–1.06)	< 0.01
**Race** (ref non-Hispanic White)				0.04		0.03		0.04
non-Hispanic Black			0.84 (0.62–1.15)		0.83 (0.61–1.13)		0.82 (0.60–1.12)	
Other			^a^0.66 (0.48–0.90)		^a^0.65 (0.47–0.89)		^a^0.66 (0.47–0.91)	
**Education level** (Increase 1 level)			0.93 (0.84–1.03)	0.15	0.97 (0.88–1.08)	0.58	1.00 (0.90–1.10)	0.93
**Insurance coverage** No (ref Yes)			0.67 (0.32–1.43)	0.30	0.74 (0.35–1.57)	0.43	0.69 (0.31–1.52)	0.36
**Lifestyle Factors**								
Physical activity Moderate to Vigorous (ref none)					^a^0.51 (0.38–0.69)	< 0.01	^a^0.52 (0.37–0.74)	< 0.01
Smoking status Former or Current Smoker (ref none)					^a^1.38 (1.09–1.76)	< 0.01	^a^1.35 (1.05–1.74)	0.02
Drinking status Moderate or above Drinker (ref none)					^a^0.61 (0.45–0.81)	< 0.01	^a^0.59 (0.43–0.82)	< 0.01
**Glycemic Control**								
Hemoglobin A1c (%) (Increase 1%)							^a^1.09 (1.01–1.18)	0.03
**Comorbidity**								
Hypertension (Ref No Hypertension)							2.00 (0.71–1.42)	0.99
Heart disease (Ref No Heart Disease)							^a^1.76 (1.33–2.34)	< 0.01
Stroke (Ref no Stroke)							0.99 (0.64–1.53)	0.96
Cancer (Ref No Cancer)							^a^1.44 (1.04–2.02)	0.03

^a^Indicates significant hazard ratio

## Discussion

In a national sample of adults with CKD and diabetes (representing 7.6 million US residents) we found that social determinants of health are significantly associated with mortality. Using a cumulative score, we found that after adjusting for relevant covariates, social determinants have a cumulative influence on mortality, with each unit increase associated with a 41% higher risk of death. In addition, after evaluating each component of the score, we found that depression is independently associated with mortality and may be of particular importance for future interventions.

While evidence on the association between social determinants of health factors and mortality exists, this is the first study to our knowledge to examine the cumulative contribution of social determinants of health factors to mortality risk in U.S. adults. Our findings have significant contributions to the literature and suggests that: 1) the effect of social determinants factors is cumulative, 2) the presence of any social determinant factor is overall detrimental and, 3) specific social determinants factors such as poverty and depression may be particularly detrimental in individuals with CKD and diabetes. The majority of studies on individual social determinant factors have examined health outcomes other than mortality [13–20, 22, 23, 25] however, our findings are comparable to the few studies on individual social determinants and mortality in people with CKD with or without diabetes [21]. For example, a study by Fedewa et al., using the Reasons for Geographic and Racial Differences in Stroke (REGARDS) cohort found that among 2761 adults with CKD stage 3 and 4, low income, a proxy for poverty, compared to high income was associated a 58% higher risk of death after adjusting for demographic, CKD stage, comorbidity and county-level poverty [21]. The exact pathway from poverty to CKD remains unclear. It is thought that poverty leads to CKD via lack of access to care, residence in food deserts, environmental exposures, disability, poor CKD risk factor control etc., and that CKD leads to poverty via disability, unemployment and health expenditures [35–37]. Furthermore, the potential for a bidirectional relationship has also been implicated [38].

The relationship between depression and mortality is established in individuals with diabetes [25, 28] or CKD with [29] or without diabetes [27] which is consistent with the results of our study. However, less work has investigated the relationship between food insecurity and mortality. In contrast to our study, a study by Walker et al., examined the relationship between food insecurity and mortality in US adults, and found that very low food insecurity was associated with a 46% higher risk of death and this relationship was not explained by comorbid diabetes and cardiovascular disease [24]. However, when food insecurity was dichotomized as food secure vs. food insecure like we did in this study, although food insecurity was initially significantly associated with mortality this significance was lost in the fully adjusted model (adjusting for demographics, comorbidities, lifestyle variables and body mass index) [24]. It is possible that the difference in study population, general population vs individuals with CKD and diabetes, could explain the differences observed. It is also possible that the way food insecurity is categorized may explain differences observed, suggesting more research is needed on the way food insecurity manifests and influences long term health outcomes such as mortality.

Our study findings have both policy and research implications. These findings highlight the importance of early recognition of adverse social determinants of health and the need to integrate screening for social determinants into clinical practice. In addition, policies and programs geared towards supporting sustainable interventions addressing multiple social determinants factors in general and in individuals with CKD and diabetes are of the essence.

Our study has some limitations that should be mentioned. First, it is a cross-sectional design so we cannot discuss causality. While there is follow-up on the individual participants on the mortality outcome, the NHANES study by design is a cross-sectional study as new individuals are interviewed each cycle, and the only data that exists in a longitudinal fashion is date of death. Second, we did not control for medication use, some of which have been shown to impact mortality risk. Third, although we controlled for multiple confounding variables, we are unable to completely rule the possibility of residual confounding. For example, the duration and severity of diabetes was not available in our dataset, even though these factors are independently associated with mortality [39, 40].

## Conclusions

In conclusion, in a national sample of adults with CKD and diabetes we found that every unit increase in cumulative social determinant of health score was associated with increased mortality. We also found that particular social determinant of health factors, such as depression, are independently associated with mortality in this population. These findings suggest that interventions are needed to address social determinant factors in individuals with CKD and diabetes.

## Data Availability

The datasets analyzed for the current study are available in the National Center for Health Statistics, https://www.cdc.gov/nchs/nhanes/index.htm

## Abbreviations

CI: Confidence Interval
CKD: Chronic kidney disease
CKD-EPI: Chronic kidney disease epidemiology collaboration
eGFR: estimated Glomerular Filtration Rate
ESRD: End stage renal disease
HbA1c: Hemoglobin A1c
HR: Hazard ratio
KDIGO: Kidney disease: improving global outcomes
NDI: National Death Index
NHANES: National Health and Nutrition Examination Survey
PHQ-9: Patient health questionnaire-9
REGARDS: Reasons for geographic and racial differences in stroke
SDOH: Social determinant of health
